# Chronic caffeine consumption curbs rTMS-induced plasticity

**DOI:** 10.3389/fpsyt.2023.1137681

**Published:** 2023-02-22

**Authors:** Megan Vigne, Jamie Kweon, Prayushi Sharma, Benjamin D. Greenberg, Linda L. Carpenter, Joshua C. Brown

**Affiliations:** ^1^Neuromodulation Research Facility, TMS Clinic, Butler Hospital, Providence, RI, United States; ^2^Department of Psychiatry and Human Behavior, Warren Alpert Medical School of Brown University, Providence, RI, United States; ^3^Department of Neurology, Warren Alpert Medical School of Brown University, Providence, RI, United States

**Keywords:** plasticity, transcranial magnetic stimulation, caffeine, long-term potentiation, d-cycloserine, motor evoked potentials

## Abstract

**Background:**

Caffeine is a widely used psychostimulant. In the brain, caffeine acts as a competitive, non-selective adenosine receptor antagonist of A1 and A2A, both known to modulate long-term potentiation (LTP), the cellular basis of learning and memory. Repetitive transcranial magnetic stimulation (rTMS) is theorized to work through LTP induction and can modulate cortical excitability as measured by motor evoked potentials (MEPs). The acute effects of single caffeine doses diminish rTMS-induced corticomotor plasticity. However, plasticity in chronic daily caffeine users has not been examined.

**Method:**

We conducted a *post hoc* secondary covariate analysis from two previously published plasticity-inducing pharmaco-rTMS studies combining 10 Hz rTMS and D-cycloserine (DCS) in twenty healthy subjects.

**Results:**

In this hypothesis-generating pilot study, we observed enhanced MEP facilitation in non-caffeine users compared to caffeine users and placebo.

**Conclusion:**

These preliminary data highlight a need to directly test the effects of caffeine in prospective well-powered studies, because in theory, they suggest that chronic caffeine use could limit learning or plasticity, including rTMS effectiveness.

## 1. Introduction

Caffeine is a ubiquitous psychostimulant which functions as a competitive, non-selective adenosine receptor antagonist of A1 and A2A subtypes. A1 receptor antagonism has been demonstrated to strengthen synapses through long-term potentiation (LTP), while A2A antagonism attenuates LTP (1). Repetitive transcranial magnetic stimulation (rTMS) is a treatment tool for neuropsychiatric disorders theorized to work through LTP, as demonstrated by animal and human work (2–6). TMS can also be paired with electromyography (EMG) recordings to measure corticomotor excitability *via* motor evoked potentials (MEPs). MEPs can detect changes in excitability when collected before and after stimulation, and are widely considered to reflect underlying plasticity, such as LTP (7, 8). In this context, TMS can probe the role of caffeine on human brain plasticity.

Notwithstanding the beneficial effects of caffeine for memory rescue (9) and LTP (1), two human studies suggest that acute caffeine intake may diminish or reverse the LTP-like after-effects of brain stimulation protocols (quantified from pre-post changes in peak-to-peak MEP amplitude). In one study using quadripulse TMS, a subset of subjects (with unknown caffeine habits) experienced robust potentiation in response to stimulation with placebo but had blunted responses with 200 mg of caffeine plus quadripulse stimulation (10). Using a different method, a separate study combined espresso administration (caffeine dose and not reported) transcranial alternating current stimulation (tACS). People not regularly consuming caffeine were randomized to caffeinated or decaffeinated espresso in a crossover design and were compared to a separate group who received no espresso. Participants in the control group (no espresso) showed the expected tACS-induced facilitation, while decaffeinated espresso blunted facilitation, and caffeinated espresso actually reversed it (11). Whether these opposing effects on LTP-like facilitation are mediated by the A2A receptor or downstream effects is not defined. Perhaps of greater clinical relevance, but still unanswered, is how chronic caffeine consumption might affect human neural plasticity or clinical rTMS effectiveness. Based on the clinical data from chronic caffeine use (9), we hypothesized that chronic caffeine users would have enhanced LTP-effects induced through our plasticity protocol relative to non-caffeine users.

## 2. Materials and methods

We performed a *post hoc* covariate analysis on self-reported caffeine-users (*n* = 16) vs. non-users (*n* = 4) from two previously published randomized crossover studies testing the LTP-like effects of a plasticity protocol combining 10 Hz rTMS and n-methyl-d-aspartate (NMDA) receptor partial agonist, d-cycloserine (DCS) (2, 5). Twenty healthy subjects (10 female, ages 21-39) were asked about their caffeine use including average number of servings per day, and on experimental day, for caffeinated soda, coffee, tea, and/or, caffeine pills (Table 1). Exclusion criteria included a recent history of psychiatric illness, use of psychotropic medication, age outside of the 18-55 range, or any contraindications to TMS, such as metal in the head or a history of seizure. A breakdown of demographic information can be found in Table 2. We also asked participants about their alcohol use habits, and these details can be found in the Supplementary material (Supplementary Table 1).

**TABLE 1:** Characteristics of caffeine users.

Caffeine use among regular users				
**Subject**	**Caffeine type**	**Amount/Day**	**Amount visit 1**	**Amount visit 2**
1	Coffee	2	1	1
2	Soda	5	3	2
3	Coffee and Tea	3	1	1
4	Coffee	2	1	1
5	Coffee and Tea	2	1	1
7	Coffee	1	1	1
8	Coffee and Tea	4	2	2
11	Coffee	1	0	0
12	Coffee	3	2	2
13	Coffee	1	1	2
14	Soda and Caffeine Pills	1	1	1
16	Coffee and Tea	1	0	0
17	Coffee	2	2	2
18	Coffee	2	1	1
19	Coffee	1	1	1
20	Coffee and Tea	2	0	0

Self-reported caffeine use among regular caffeine users. Subjects 6, 9, 10, and 15 did not use caffeine regularly, and are thus excluded. Daily average intake (reported as number of servings) ranged from 1 to 5 caffeine servings per day. Participants reported caffeine use before each visit. Caffeine modalities included coffee, tea, caffeinated soda, and caffeine pills.

**TABLE 2:** Demographic characteristics.

	Caffeine group *n* = 16	Non-caffeine group *n* = 4
Age, M (SD)	29.1 (5.0)	25.3 (3.2)
Sex assigned at birth, N (%) female	7 (0.44)	3 (0.75)
**Racial identity, N (%)**		
Asian	3 (0.19)	0 (0.0)
Black or African American	1 (0.06)	0 (0.0)
More than one race	0 (0.0)	1 (0.25)
White	10 (0.63)	3 (0.75)
Pacific islander	1 (0.06)	0 (0.0)
**Ethnic identity, N (%)**		
Hispanic/Latino	1 (0.06)	1 (0.25)
Chinese	0 (0.0)	1 (0.25)
Caucasian	9 (0.56)	2 (0.5)
Chamorro	1 (0.06)	0 (0.0)
Indian	3 (0.19)	0 (0.0)
Romanian	1 (0.06)	0 (0.0)
Nigerian	1 (0.06)	0 (0.0)

Age, sex, race, and ethnicity information for all 20 participants. Race information was not reported for one participant.

In each study, the participants received a single dose of 100 mg d-cycloserine (DCS), or microcrystalline cellulose placebo (PBO) capsule, at least 1 week apart. Detailed methods of TMS procedures can be found elsewhere (2, 5). Briefly, all TMS procedures were neuronavigated (Brainsight, Rogue Research, Quebec, Canada) to the left primary motor cortex (M1) using a template brain. Single pulses were jittered every 4-7 s, collected into bins of 20 pulses and averaged. Averages after rTMS were normalized to pre-rTMS baseline within each subject. 10 Hz rTMS was delivered at 80% of resting motor threshold for 1.5 s on, 58.5 s off for 20 min (300 pulses). DCS was given 1 h before SP measures and 2 h before rTMS.

Because our small sample size cannot be assumed to be normally distributed, we used non-parametric statistical methods. Normalized MEP averages between all four groups [drug condition (placebo vs. DCS) and group (caffeine vs. non)] across time and for each individual time point were compared with Kruskal-Wallis tests. Between-subject comparisons (caffeine vs. non) were analyzed with Mann-Whitney U tests. Within-subject comparisons between drug conditions were analyzed with Wilcoxon Signed-Rank tests. Analyses were performed with SPSS for MAC (version 28.0.1.0, IBM Corp., NY, USA). *A priori* level of significance was set at *p* < 0.05.

## 3. Results

**FIGURE 1:**
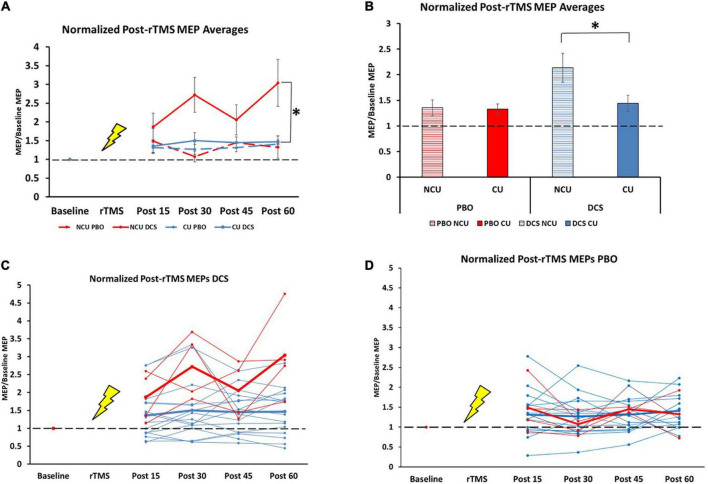
Difference in response to plasticity protocol for caffeine users and non-users. NCU = non-caffeine users, CU = caffeine users, *n* = 20 (Mean ± SEM). **(A)** Normalized post-rTMS MEP averages for all groups at all timepoints. 15 min: NCU PBO (1.48 ± 0.33); CU PBO (1.32 ± 0.15); NCU DCS (1.88 ± 0.36); CU DCS (1.36 ± 0.17) [H (3) = 1.8, *p* = 0.61]. 30 min: NCU PBO (1.07 ± 0.14); CU PBO (1.27 ± 0.13); NCU DCS (2.72 ± 0.47); CU DCS (1.50 ± 0.21) [H (3) = 8.51, *p* = 0.037]). 45 min: NCU PBO (1.45 ± 0.03); CU PBO (1.31 ± 0.11); NCU DCS (2.06 ± 0.40); CU DCS (1.45 ± 0.15) [H (3) = 2.85, *p* = 0.42]. 60 min: NCU PBO (1.33 ± 0.30); CU PBO (1.41 ± 0.11); NCU DCS (3.04 ± 0.63); CU DCS (1.47 ± 0.16) [H (3) = 7.1, *p* = 0.07]. **(B)** Normalized post-rTMS MEPs grand averages across 1 h time course. NCU PBO (1.35 ± 0.15); CU PBO (1.33 ± 0.10); NCU DCS (2.14 ± 0.28); CU DCS (1.44 ± 0.16). **(C)** Individual (thin lines) and group average (thick lines) normalized MEPs in DCS condition for NCU (red) and CU (blue). **(D)** Individual (thin lines) and group average (thick lines) normalized MEPs in PBO condition for NCU (red) and CU (blue). *Indicates significance at the 0.001 level.

There were no differences in baseline MEP amplitude between the four conditions [H (3) = 3.15, *p* = 0.37]. We observed an overall effect of our plasticity protocol (DCS + 10 Hz rTMS) over 1 h between the four conditions {drug [DCS and placebo] and status [non-caffeine users (NCU) and caffeine users (CU)]}, [H (3) = 18.7, *p* < 0.001; Figures 1A, B]. Specifically, NCU had greater potentiation than CU in the DCS condition [U (N_Caffeine_ = 63, N_Non–caffeinne_ = 16] = 200, *z* = −3.7, *p* < 0.001; Figures 1A, C), whereas no differences between NCU and CU were observed in the PBO condition [U (N_Caffeine_ = 61, N_Non–caffeinne_ = 15] = 452, *z* = −0.072, *p* = 0.94; Figures 1A, D). Within-subject comparisons by drug condition revealed NCU subjects had greater facilitation with DCS than with PBO (*T* = 107, *z* = −2.7, *p* = 0.008). In contrast, no differences between DCS and PBO conditions were observed in CU (*T* = 936, *z* = −0.16, *p* = 0.88).

## 4. Discussion

Contrary to our hypothesis that chronic caffeine would enhance LTP-like plasticity, we found that DCS combined with 10 Hz rTMS produced robust MEP facilitation in non-caffeine users (NCU), which was blunted in caffeine-users (CU) to a level seen in the placebo condition for both groups (see Figure 1A). These data suggest that chronic caffeine users could have decreased capacity for LTP-like plasticity. The effects we found are not accounted for by changes in baseline excitability, as these did not differ between caffeine-users and non-users, consistent with previous findings (12). To our knowledge, there are no known direct interactions between caffeine and NMDA or d-cycloserine to explain these effects either.

It is important to note that conclusions from this study remain preliminary as there were only four subjects in the NCU group (vs. 16 in the CU group), thus likely overestimating the effect size. The imbalance between CU and NCU groups reflects an inherent weakness in our *post hoc* covariate analyses. Future studies may resolve this limitation by selectively recruiting matched (i.e., age, education, socioeconomics) CU vs. NCU in well-powered studies, which would also retain real-world relevance pertaining to actual caffeine consumption habits – a relative strength of this analysis. Additionally, disentangling acute from chronic caffeine effects is needed, as we observed similar effects to acute caffeine studies (10, 11). This could be assessed with a prospective 3-arm study including chronic + acute, chronic–acute, and acute–chronic conditions. Prospectively testing caffeine use may not be feasible in human research, but by aligning protocols with animal studies, conclusions about causality and underlying mechanisms may be reached. Furthermore, caffeine/plasticity dose-response relationships could strengthen conclusions and may provide causality insights in a well-powered study. Our analysis relied on self-reported dosages, consistency, and regularity of naturalistic caffeine use. Therefore, we cannot create a dose-response curve based on caffeine bioavailability at the time of experimentation. However, we estimated an average daily consumption of 137 mg/day, with a range of 30-270 mg/day by using averages reported in previous trials (90 mg/serving of coffee, 30 mg for tea, 30 mg for soda, 80 mg for energy drinks, and 200 mg for caffeine pills) (13, 14). This limitation could be managed and precision could be improved with the evaluation of caffeine serum concentration correlations with caffeine dose and timing, lending a better estimate of CNS bioavailability and correlation with plasticity responses.

Bearing these limitations in mind, we will briefly speculate on the meaning and potential mechanisms of these findings. It is tempting to conclude that the blunted plasticity observed in caffeine users may be the result of non-selective antagonism of the A_2A_ receptor, as A_2A_ antagonism attenuates LTP (1), and this LTP-mitigating effect is eliminated in A_2A_ receptor knockout mice and with selective antagonists (15). On the other hand, and similar to our findings, chronic caffeine administration to rats markedly diminished LTP over 48 h of *in vivo* hippocampal recordings (16). Unfortunately, the overall effects of caffeine are not so clear-cut, with opposing directional outcomes still unreconciled (17). Some insight may be gained through closer examination of the underlying mechanisms. Caffeine is known to mobilize intracellular calcium (18, 19). Differential calcium concentrations have opposing effects on plasticity; with chronic low-levels of calcium leading to LTD, and acute high-levels leading to LTP (3). Indeed, differential calcium levels are proposed to also underlie opposing effects of plasticity-inducing brain stimulation protocols (20). Whereas we would expect LTP with acute caffeine, we are looking at chronic intake, likely leading to modest calcium levels in between the levels of LTD and LTP, furthering complicating our ability to make speculations about the interplay of intracellular calcium and plasticity as a function of caffeine and rTMS. Regardless, this common factor may hint at a possible mechanistic basis for caffeine’s effect on LTP-like aftereffects in brain stimulation protocols. The durability of rTMS-mediated plasticity is of great interest, but rarely assessed. In both classical LTP experiments (21) as well as human MEP experiments (22, 23) it is common to measure responses up to 30 min. While we measured only up to 1 h, several non-invasive brain stimulation studies have noted LTP-like after-effects lasting more than 24 h following stimulation (24–26). These changes may correspond with AMPA receptor insertion and spine expansion seen in animal models for several hours (6).

In conclusion, we aimed to evaluate the naturalistic differences between chronic caffeine users and non-users in response to an excitatory plasticity-inducing brain stimulation protocol. Our data suggests that while non-caffeine users have a robust facilitation, chronic caffeine use blunts plasticity. Importantly, our conclusions are limited by naturalistic study design and a small number of non-caffeine users. Nevertheless, these results may guide future study design and dosage considerations. Perhaps a more pressing question is whether caffeine inhibits clinical rTMS responses proposed to work through LTP-like mechanisms. This question remains unanswered as our experiment varied from clinical rTMS in several important ways: we administered a single session with fewer pulses over the motor cortex of healthy subjects, in contrast with clinical rTMS which delivers ten times the pulse number over 36 sessions to the prefrontal cortex of depressed brains. Notwithstanding these differences, data exists supporting the relevance of MEP plasticity measures in clinical rTMS, with those having greater MEP plasticity before rTMS were more likely to respond clinically (27, 28). Like clinical TMS outcomes, there is marked interindividual variability observed in human neurophysiology measures (i.e., MEPs) (29); caffeine may account (and provide a mechanistic basis) for some of this variability and may account for a portion of the enhancement seen with DCS in our pilot studies (2, 5). While the difference between caffeine users and non-users appears robust in this analysis, the non-users represent only a small proportion of subjects. Robust effects of only a few subjects may explain the smaller effect size seen in the aforementioned studies. Alternatively, confounding variables that may accompany no caffeine habits have not been ruled out, and in theory, could explain enhanced plasticity. For example, it is possible that individuals with innately higher plasticity (for other reasons) are less drawn to caffeine, and that these innate properties are driving the overall difference in the earlier studies as well as our findings here. While the effect of chronic daily caffeine use remains unclear, but considering the pervasiveness of caffeine use, a better understanding of how caffeine alters the underlying mechanism of learning and memory, as well as the potential impact of caffeine on clinical rTMS effects, merits further attention.

## Data availability statement

The raw data supporting the conclusions of this article will be made available by the authors, without undue reservation.

## Ethics statement

The studies involving human participants were reviewed and approved by Butler Hospital Institutional Review Board. The patients/participants provided their written informed consent to participate in this study.

## Author contributions

MV conducted the data collection, literature review, statistical analysis, and writing the manuscript. JK assisted with the data collection, statistical analysis, and manuscript preparation. PS assisted with manuscript preparation. JB designed the study and protocol and assisted with analysis and manuscript edits. BG and LC assisted with study and protocol design, as well as review of the manuscript. All authors contributed to the article and approved the submitted version.
