# Supplementary material for: Chronic caffeine consumption curbs rTMS-induced plasticity
Source: Front Psychiatry. 2023 Feb 22;14:1137681. doi: 10.3389/fpsyt.2023.1137681 (PMC9993245; doi:10.3389/fpsyt.2023.1137681)
Supplement: Supplementary file 1 [file Table_1.pdf]

Supplementary Material

1.1 Supplementary Tables

Supplementary Table 1. Alcohol use habits of self-reported alcohol drinkers. Self-reported alcohol use among regular alcohol consumers. Subjects 2, 4, 6, 9, and 14 did not use alcohol, and are thus excluded. Types of alcohol included beer, wine, and liquor. Participants reported the average number of servings consumed on an occasion of drinking, not daily intake. Frequency of alcohol use was also self-reported. NR represents no response.

Subject ID	Alcohol Type	Amount	Frequency
1	Beer	1	1-2/Day
3	Beer, Wine	1	3-4/Week
5	Wine	0	1-2/Six Months
7	Beer, Wine, Liquor	1	NR
8	Beer, Wine, Liquor	2	Most Weekends
10	Wine	2	4/Week
11	Beer	NR	NR
12	Beer, Wine, Liquor	NR	1-2/Week
13	Beer	0.5	NR
15	Wine	1	1/Week
16	Liquor	1	NR
17	Beer	1	NR
18	Wine	2	NR
19	Beer, Wine, Liquor	0	NR
20	Wine, Liquor	1-2	NR
